# ‘Isn’t it ironic?’ Beliefs about the unacceptability of emotions and emotional suppression relate to worse outcomes in fibromyalgia

**DOI:** 10.1007/s10067-017-3590-0

**Published:** 2017-03-02

**Authors:** Hannah Bowers, Abigail L. Wroe, Tamar Pincus

**Affiliations:** 1 Psychology Department, Royal Holloway University of London, Egham, TW10 0EX UK; 2 Department of Clinical Psychology, Royal Holloway University of London, Egham, UK

**Keywords:** Beliefs about emotions, Emotional suppression, Fibromyalgia, Support-seeking

## Abstract

Beliefs about the unacceptability of experiencing and expressing emotions have been found to be related to worse outcomes in people with persistent physical symptoms. The current study tested mediation models regarding emotional suppression, beliefs about emotions, support-seeking and global impact in fibromyalgia. One hundred eighty-two participants took part in an online questionnaire testing potential mechanisms of this relationship using mediation analysis. The model tested emotional suppression and affective distress as serial mediators of the relationship between beliefs about emotions and global impact. In parallel paths, two forms of support-seeking were tested (personal/emotional and symptom-related support-seeking) as mediators. Emotional suppression and affective distress significantly serially mediated the relationship between beliefs about emotions and global impact. Neither support-seeking variable significantly mediated this relationship. Results indicate a potential mechanism through which beliefs about emotions and global impact might relate which might provide a theoretical basis for future research on treatments for fibromyalgia.

## Introduction

Fibromyalgia is a condition consisting of widespread pain, tender points, fatigue, sleep disturbance and mood problems [1]. It occurs in 2.2–6.4% of the US population [2] and 1.7–5.4% in UK samples [3]. It is more common in women than in men [3]. Some evidence has supported central nervous system dysfunction as a possible aetiology of fibromyalgia. In particular, participants with fibromyalgia have shown a reduced pressure pain threshold compared to controls which has been explained by descending analgesic activity as well as central sensitisation [4]. Despite this evidence, there remains uncertainty about aetiology and factors which may contribute to the impact the disorder has on a person’s life, with some evidence looking into psychological variables which may relate to outcomes of the disorder.

Research has investigated potential psychological mechanisms involved in the development and maintenance of persistent physical symptoms, particularly those with aetiological uncertainty. Such biopsychosocial models include interrelating psychological variables that work to maintain symptoms [5]. For chronic pain, fear avoidance has been highlighted where fear and consequent avoidance of activity results in increased pain and disability through deconditioning [6]. Prior research has also established a role of cognition in the maintenance of fibromyalgia whereby negative interpretations of pain can result in increased fear of pain. This in turn leads to somatic hypersensitivity [7]. Much of the research into psychological aspects of pain has been focussed on chronic pain as a whole and the transition from acute to chronic pain more generally as opposed to focussing solely on fibromyalgia. This is despite evidence that fibromyalgia patients are distinct from other chronic pain patients on factors such as psychological distress [8] and the role of distress in pain responses [9]. Thus, investigations into the psychological features of fibromyalgia specifically, as opposed to pain more generally, are warranted.

In line with evidence on avoidance of activity, avoidance of emotions has also been studied. Other researchers have investigated emotion regulation in fibromyalgia patients and found that there is more emotional suppression in those with fibromyalgia compared to healthy controls [10]. In the same study, emotional expression was negatively correlated with fatigue, however not with pain. Similar findings regarding emotional expression come from written emotional disclosure interventions in fibromyalgia, with evidence that writing expressively about one’s emotions results in improvements in global impact, health care utilisation, disability, pain, fatigue and psychological well-being [11, 12].

The benefits of emotional expression could be explained with regards to ironic processing effects where the suppression of undesirable thoughts and feelings results in an ironic increase in that particular feeling [13]. Thus, emotional suppression might result in increased negative affect which may then have an impact on the person’s life with regards to their disorder [14].

More recently, there is a focus on emotional suppression in psychological therapy for people with persistent physical symptoms. For example, Acceptance and Commitment Therapy (ACT) focusses on experiential avoidance of unpleasant sensations, such as pain and emotions, and moving towards acceptance of such sensations [15]. There is evidence for the effectiveness of ACT and Mindfulness [16] in people with persistent pain with respect to distress. A meta-analyses of treatments of fibromyalgia concluded that cognitive behavioural therapy (CBT) is superior to other psychological treatment methods [17]. The Cochrane review of chronic pain treatments (including fibromyalgia) suggests the need for further investigation into possible cognitive and behavioural mechanisms of treatment, stating there is a need for better theory-driven hypotheses of the mechanisms of change in treatments for pain [16].

Cognitive behavioural therapy supports individuals in understanding maintaining cycles in relation to thoughts, feelings, behaviours and bodily symptoms and making changes to respond in more helpful ways. Evidence suggests CBT is a useful intervention for fibromyalgia and that the mechanisms (e.g. changes in beliefs) warrant further investigation in order to provide more theoretically driven interventions [16, 17]. Previous research, focussed on improving our understanding of thought and behaviour patterns that may be related to persistent physical symptoms, demonstrated that emotional suppression is associated with beliefs about the unacceptability of experiencing emotions [18]. The role of such beliefs has been demonstrated in patients with chronic fatigue syndrome (CFS), where beliefs about the unacceptability of emotions were higher in patients with CFS compared to healthy controls, and these beliefs were related to increased fatigue [19]. Similarly in irritable bowel syndrome (IBS), beliefs about the unacceptability of emotions were related to reduced quality of life and participants with IBS scored higher on the Beliefs about Emotions Scale compared to healthy controls [18].

Previous cross-sectional research in IBS found that suppression alone did not mediate the relationship between these particular beliefs about emotions and quality of life, suggesting a need to investigate other potential mediators of this relationship [18]. In line with ironic processing effects, distress should be investigated within this model. A further explanation proposed for the relation between beliefs about the unacceptability of emotions and outcomes is that suppression of emotion may result in a reduction in social support [19]. That is, if one is unwilling or unable to express their unpleasant emotions to their support network, that network will be unable to offer support. Both potential mechanisms warrant further investigation to explore the role of emotional suppression in fibromyalgia.

The current study therefore aimed to improve our understanding of the role of beliefs about the unacceptability of emotions and emotional suppression in fibromyalgia. In particular, the study aimed to investigate three possible indirect mediation paths for the relationship between beliefs about emotions and global impact in fibromyalgia. The first indirect effect consisted of two serial mediators: emotional suppression and affective distress. This first path tested the ironic processing explanation that maladaptive beliefs about emotions and consequent emotional suppression will result in poorer outcomes via an ironic increase in the emotions one is attempting to suppress. An alternate model with the two mediators inverted was also tested to evaluate the proposed direction of this indirect effect.

Two further paths were tested to assess support-seeking as a potential mediator of the relationship between beliefs about emotions and global impact in fibromyalgia. This is in line with the supposition that believing the expression of emotions to be unacceptable will result in a reduction in social support-seeking and thereby impact the individual’s life [20]. Both emotional support-seeking and symptom-related support-seeking were tested in parallel.

It is hypothesised that the relationship between beliefs about emotions and global impact will be significantly mediated by emotional suppression and affective distress in a serial manner. It is also predicted that in parallel, personal/emotional and symptom-related support-seeking will mediate this relationship. It is hypothesised that the alternate model will not be significant.

## Materials and methods

### Diagnostic criteria

Participants completed the London Fibromyalgia Epidemiology Study Screening Questionnaire [20]. To be included, participants were required to have pain in muscles, bones or joints, lasting at least 3 months. To be considered to have fibromyalgia, they must have had pain in their shoulders, arms or hands, legs or feet and neck, chest or back. In addition to these criteria, to be considered to have fibromyalgia, one of the following must apply:

1. Pain in shoulder, arms or hands is on both sides and pain in legs or feet is on both sides.
2. Pain in shoulder, arms or hands is on the right side and pain in legs or feet is on the left side.
3. Pain in shoulder, arms or hands is on the left side and pain in legs or feet is on the right side.

Further to these criteria, participants must state that a physician has diagnosed them with fibromyalgia. If participants did not meet the criteria but stated a diagnosis of fibromyalgia, they were not included in the analysis. If a participant stated they have another diagnosis which might better explain these symptoms, they were also excluded. Those with comorbid conditions were not excluded from the analysis so as to represent the complex nature of fibromyalgia, which includes frequent comorbidities such as CFS [21] and arthritis [22].

### Participants

Participants were recruited online through websites and forums dedicated to fibromyalgia (including Reddit and Facebook discussion forums). Of the 212 participants who took part, 194 met the criteria for fibromyalgia. Sixteen participants claimed to be diagnosed with fibromyalgia but did not meet the criteria, and three participants said they had another disorder which would better account for the symptoms in the screening tool. One participant did not provide sufficient data to be analysed for path 1 (with emotional suppression and affective distress as mediators). Additionally, due to missing data in support-seeking responses, 183 participants had sufficient data for testing paths 2 and 3 (emotional and symptom-related support-seeking respectively) and therefore made up the final sample (174 females, mean age (SD) = 46.99 (11.84). Participants provided demographic information (age, sex, employment status, educational level, ethnicity and country of birth) prior to completing the questionnaires (see Table 1). The current study received ethical approval from the university’s departmental ethics committee.

**Table 1:** Demographic information of participants included in the final model

Variable	*N* (%)	
Age: mean years (S.D)	44.67	(17.96)
Sex		
Female	174	(95.6)
Ethnicity		
Caucasian	176	(96.7)
Mixed White and Black Caribbean	1	(0.5)
Mixed White and Asian	1	(0.5)
Other mixed ethnic background	2	(1.1)
Black/African/Caribbean	2	(1.1)
Highest education level		
Senior/secondary school (up to 16)	15	(8.2)
Sixth form/college (up to 18)	24	(13.1)
University undergraduate	37	(20.3)
University postgraduate	21	(11.5)
Employment		
In paid employment	81	(44.5)

### Measures

#### Beliefs about Emotions Scale

Beliefs about the unacceptability of the experience and expression of emotions were measured using the Beliefs about Emotions Scale (BES) developed by Rimes and Chalder [19]. Participants rated on a seven-point scale their agreement with 12 items, such as “it is a sign of weakness if I have miserable thoughts”. A total score across items resulted in a maximum possible score of 72, where high scores demonstrate more strongly believing that expressing and experiencing emotions is unacceptable. This questionnaire has been validated in participants with CFS and was found to be internally consistent (Cronbach’s alpha = 0.91). This sample scored more highly than healthy controls, and scores were significantly related to perfectionistic beliefs and fatigue in this clinical sample [19].

#### Courtauld Emotional Control Scale

The Courtauld Emotional Control Scale (CECS) was used to measure emotional suppression [23]. Participants were asked to state how frequently a particular statement applies to them on a four-point scale from always to never. Twenty-one items covered three emotions: unhappiness, anger and anxiety. These were assessed with items such as “when I feel unhappy I bottle up” as well as negatively scored items including “when I feel angry I say what I feel”. Summing across all 21 items resulted in a maximum possible score of 84, with high scores indicating more emotional suppression.

The current study chose this measure of emotional suppression due to its clear behavioural focus, compared to other measures which also incorporate cognitive aspects of emotional suppression, such as beliefs. Furthermore, the CECS provides a clear focus on undesirable emotions whereas other subscales of existing measures do not specify particular emotions.

The CECS has been found to be valid and reliable with strong correlations between overall scores and each subscale and evidence of internal consistency (Cronbach’s alpha = 0.88) [23]. There was good test-retest reliability in this measure, and it has been validated in hospital employees, cardiac patients and breast cancer patients [23, 24].

#### Hospital Anxiety and Depression Scale

The Hospital Anxiety and Depression Scale (HADS) [25] was used to measure affective distress. Fourteen items ask participants to rate on varying four-point scales their level of depression and anxiety symptoms, with seven items addressing each affect. Scores were totalled across both depression and anxiety items to create one overall affective distress score with a maximum of 42. Overall HADS scores across the two subscales have been used in a range of samples including those in primary care [26] and those with musculoskeletal pain [27].

HADS was chosen due to its deliberate exclusion of somatic symptoms of anxiety and depression. This exclusion is considered crucial in participants with physical conditions to truly and validly capture the affective aspects of depression and anxiety without any physical aspects of their condition confounding scores and has therefore been used as a reliable measure of affective distress in clinical populations (Cronbach’s alphas = 0.82–90) [28].

#### General Help-Seeking Questionnaire

The General Help-Seeking Questionnaire (GHSQ) measures help-seeking intentions across two questions. For each question, the participant is asked to rate how likely (on a seven-point scale) they would ask a particular person for help. Each of the two questions contained five possible people the individual might seek help from: intimate partner, friend, parent, other relative/family member and minister/religious leader. Since the current study was interested in social support-seeking and not formal support-seeking, medical and mental health professionals were removed. Scores were summed for each participant giving a maximum possible score of 35.

In the original version, the first question asks from whom one would seek help if experiencing personal/emotional problems. The second question asks the same but in relation to suicide ideation. The authors of the GHSQ recommended modification to suit the particular samples being studied. Therefore, for question 2, the current study asked about support-seeking in relation to “particularly troublesome symptoms of fibromyalgia”. In addition, for the purpose of the current study, the word “help” was replaced with “support” so as to encourage reflection of emotional support as opposed to focussing more on physical or practical support.

The GHSQ had been found to be reliable and valid, with good internal consistency (Cronbach’s alpha = 0.85) and test-retest reliability (*r* = .92). During development, the scale was validated in high-school students and found that scores were significantly correlated with self-reported help-seeking behaviour [29]. In the current study, symptom-related and personal/emotional support-seeking both showed good levels of internal consistency (Cronbach’s alphas = 0.77 and 0.80 respectively).

#### Revised Fibromyalgia Impact Questionnaire

The Revised Fibromyalgia Impact Questionnaire (FIQr) was used to measure global impact of fibromyalgia [30]. It measures the impact of the disorder across three domains: function, overall impact and symptoms. Participants responded to items on varying scales where 10 always indicated greater impact. Scores across the three domains were summed and converted to a score out of 100, where high scores indicate greater impact of the disorder.

There is evidence of excellent internal consistency of the FIQr (Cronbach’s alpha = 0.95), and correlations with the original FIQ were good (*r* = .65 for overall scores) as were correlations of all domains with the subscales of the Short Form (36) Health Survey [30].

### Design and statistical analysis

The current cross-sectional study employed correlational methods to assess potential indirect effects that might explain the relationship between beliefs about emotions and global impact in fibromyalgia. The indirect effects were tested using Preacher and Hayes’s Process plug-in for SPSS with bootstrapping [31]. Multiple paths were tested in one model. For an indirect effect to be significant, the 95% confidence intervals must not contain zero. An additional alternate model was tested with the serial mediators of path 1 inverted so as to test the hypothesised direction of the effect.

**Fig. 1:**
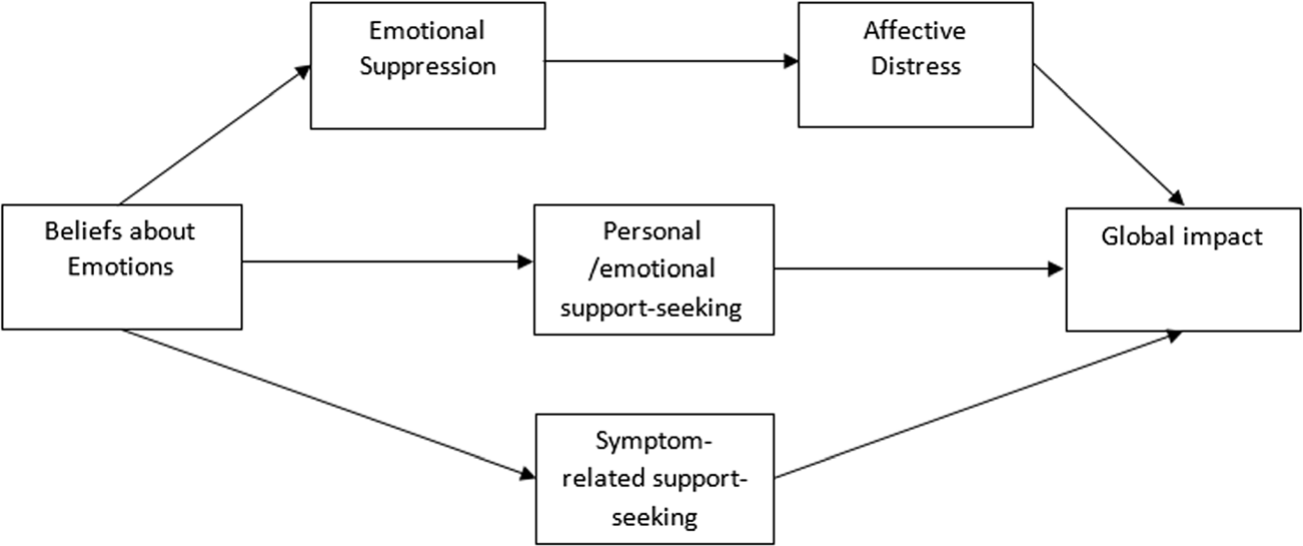
Tested paths in the mediation model

The first path tested consists of two mediators working serially: emotional suppression and affective distress. While mediation uses correlational methods, testing of serial multiple mediation models “assumes a causal chain linking the mediators, with a specific direction of causal flow” [32]. Thus, for this first path, the chain was beliefs about emotions → emotional suppression →affective distress → global impact. As all paths were tested in one model (see Fig. 1), thus this path was tested while also accounting for variance explained by paths 2 and 3.

Paths 2 and 3 consisted of the same predictor (beliefs about emotions) and outcome (global impact). For path 2, the mediator was personal/emotional support-seeking, and for path 3 the mediator was symptom-related support-seeking. As before, since all paths were tested in one model, the variance in other mediators is accounted for when testing each individual path.

The direction of this first path was further tested by inverting the two mediators in an additional alternate model to produce the following chain: beliefs about emotions → affective distress → emotional suppression → global impact. In this model (again controlling for paths 2 and 3), the predictor and outcome were the same. However, the serial order of the mediators was affective distress and then emotional suppression.

Where more than 20% of data were missing for items of a single variable, cases were excluded. Where less than 20% of data were missing, values were imputed using estimated maximisation techniques [32].

## Results

Mediation analyses were conducted with beliefs about emotions, emotional suppression, affective distress, personal/emotional support-seeking, symptom-related support-seeking and global impact in the model. Descriptive statistics for all variables in the model can be found in Table 2.

**Table 2:** Descriptive statistics of all variables in the model

Variable	Mean (S.D)
Beliefs about Emotions Scale [range 0–72] (Higher scores indicate greater unacceptability of emotions)	41.55 (15.40)
Courtald Emotional Control Scale [range 21–84] (Higher scores indicate greater emotional suppression)	59.78 (13.57)
Hospital Anxiety and Depression Scale [range 0–42] (Higher scores indicate greater affective distress)	22.75 (7.75)
General Help-Seeking Questionnaire—personal/emotional [range 5–35] (Higher scores indicate greater intention to seek help)	17.81 (7.09)
General Help-Seeking Questionnaire—symptoms [range 5–35] (Higher scores indicate greater intention to seek help)	17.05 (7.08)
Fibromyalgia Impact Questionnaire [range 0–100] (Higher scores indicate greater impact of the disorder on a person’s life)	68.31 (16.41)

### Path 1: Emotional suppression and affective distress

An indirect effect was tested where the relationship between beliefs about emotions and global impact was mediated by emotional suppression and affective distress in a serial manner. Emotional suppression and affective distress both serially mediated the relationship between beliefs about emotions and global impact (standardised indirect effect = 0.0809). The significance of this positive indirect effect was tested using bootstrapping procedures (indirect effect = 0.0862, 95%CI [0.1549, 0.1652]) and was found to be significant.

### Path 1: Alternate model

An alternate model testing the direction of path 1 was analysed in a model along with paths 2 and 3. In this model, the two serial mediators were inverted. Affective distress and emotional suppression did not serially mediate the relationship between beliefs about emotions and global impact in that order (standardised indirect effect = −0.0045). Bootstrapping procedures were used to test the significance of this indirect effect (indirect effect = −.0047, 95% CI [−0.0199, 0.0002]).

### Path 2: Personal/emotional support-seeking

Personal/emotional support-seeking was tested as a mediator of the relationship between beliefs about emotions and global impact, in parallel with paths 1 and 3. Personal/emotional support-seeking did not mediate this relationship (standardised indirect effect = −0.0009). Using bootstrapping procedures, this indirect effect was found to be non-significant (indirect effect = −0.0010, 95% CI [−0.0751, 0.0479]).

### Path 3: Symptom-related support-seeking

Symptom-related support-seeking was tested as a mediator of the relationship between beliefs about emotions and global impact, in parallel with paths 1 and 2. Symptom-related support-seeking did not mediate this relationship (standardised indirect effect = −0.0044). Using bootstrapping procedures, this indirect effect was found to be non-significant (indirect effect = −0.0047, 95% CI [−0.0402, 0.0277]).

**Fig. 2:**
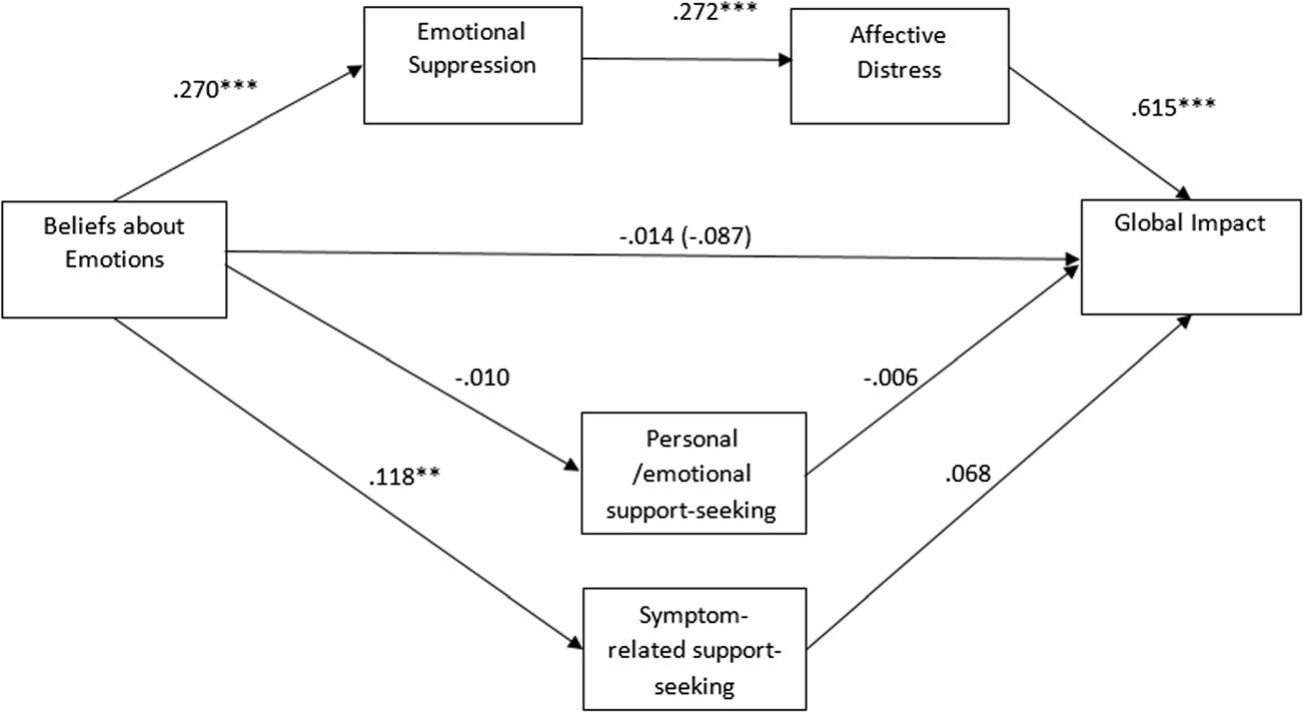
Standardised regression coefficients for the relationships between the three paths. Each path was tested while controlling for the unique variance in other included paths.*p < .05, **p < .01, ***p < .001

See Fig. 2 for coefficients of the relationships between variables.

## Discussion

The current study tested three possible indirect effects through which beliefs about emotions and global impact might relate. Path 1 tested the ironic processing theory of emotional suppression while paths 2 and 3 tested two forms of support-seeking as potential mediators. An alternate model was tested to assess the hypothesised direction of path 1.

Emotional suppression and affective distress serially mediated the relationship between beliefs about emotions and global impact in fibromyalgia. That is, an indirect effect was found that is mediated first by emotional suppression and then affective distress. This particular path is in line with ironic processing effects [13]. This theory suggests that beliefs about emotions might relate to greater impact of the disorder firstly through an increase in emotional suppression, which in turn would relate to greater affective distress. This increase in affective distress may then be associated with increased global impact. This direction is supported by a non-significant alternate model with the mediators inverted, though experimental testing is required to determine causal relationships.

This evidence supporting a significant indirect effect compliments previous evidence showing emotional expression interventions to be beneficial to patients with fibromyalgia [11, 12]. This suggests that interventions focussing on beliefs and behaviours around emotions may be helpful for people with fibromyalgia. The findings are consistent with the proposal that a mechanism of changes in outcomes around fibromyalgia may relate, at least to some extent, to changes in beliefs and behaviours around emotions. CBT might be used to support identification and evaluation of beliefs about emotions and support changes in behaviours around emotion, such as emotional suppression. ACT, which had also been shown to be helpful for people with FMS [33], may focus specifically on acceptance, for example, of emotions (as well as symptoms of pain). Further research is required to evaluate mechanisms of change, such as beliefs, behaviours and acceptance, through different psychological approaches.

While there was a significant indirect effect found in the current study, the direct relationship between beliefs about emotions and global impact was not significant. This is somewhat unexpected given that there was a significant positive indirect effect via emotional suppression and affective distress. While there may be some detriment to holding these beliefs about the unacceptability of emotions, there may also be a protective factor, thereby cancelling out any significant direct relationship. This is known as inconsistent mediation whereby multiple mediators may elicit separate indirect paths in opposing directions, resulting in a non-significant overall relationship [34]. Further research into other potential mediators and moderators of this relationship (for example, acceptance of emotions and of physical symptoms as well as other emotion processing variables such as alexithymia [35]) may help explain the inconsistent mediation found in the current study.

The roles of emotional suppression and beliefs about emotions have been established in a model through which distress appears to be a key mechanism. Some might argue that existing interventions targeting affective distress are sufficient in that they address this aspect of the model which relates to outcome measures. Effective therapy for reducing distress focusses on identifying and adapting cognitions and behaviours which are related to the experience of depression/anxiety, while interventions addressing these affective symptoms alone (e.g. medication) are less effective and cost-effective [36, 37]. Evidence in treatments for chronic pain with depression has shown that stepped care (involving anti-depressant treatment followed by a self-management pain program) elicits clinically significant improvements in both depression and pain for only 26% of patients [38]. A secondary analysis revealed that the beliefs and cognitions of participants (in particular higher levels of fear avoidance) predicted reduced response [39]. This suggests a need to focus on specific maladaptive cognitions. Future research may wish to focus on the extent to which changes in beliefs and behaviours around emotions are a mechanism of change in both CBT (which focusses specifically on identifying and evaluating beliefs and behaviours) and ACT (which focusses more on acceptance of emotions and possibly indirectly on challenging idiosyncratic beliefs).

Conversely to the hypotheses, neither support-seeking variable significantly mediated the relationship between beliefs about emotions and global impact. Support-seeking was found to be related to beliefs about emotions but not global impact. Measuring support-seeking intentions as opposed to actual received social support could explain this finding. There may be confounding variables influencing the amount of social support received which might ultimately be more closely related to impact of the disorder on a person’s life. For example, there may be possible interactions between who they ask for help and how they ask for help that have not been adequately measured in the current study. Furthermore, the current measure of help-seeking was adapted for use in the current sample and therefore was not validated. While the amended scales did show good internal consistency, their construct validity may be questioned. Future research using validated measures of support-seeking might provide evidence for a role of support-seeking in the current model.

The present study used correlational methods to test hypothesised causal paths. An alternate path was tested and was found to be non-significant thereby supporting the predicted direction for path 1. However, in order to establish a causal relationship between these variables, experimental methods with a clear timeline should be employed in future research. Thus, while the current study supports a hypothesised causal model, it does not explicitly test causality so further experimental research is needed.

The online methods used may have reduced the control within the current study. Participants recruited online might be characteristically different from those found in community and/or clinical settings. Furthermore, those who respond to online recruitment requests might differ compared to those who do not respond, thereby providing a potentially biased sample. Additionally, the use of online methods meant that clinicians’ diagnosis was self-reported which may reduce its reliability. The findings of the current study, while interesting, could be tested further in clinical samples with clinician-confirmed diagnoses.

The current study found support for the role of beliefs about emotions in fibromyalgia whereby emotional suppression and affective distress mediate its relationship with global impact, which, with further research, may then inform treatment in these samples.
